# Moss‐Accumulated eDNA Is a Promising Source for Terrestrial Biodiversity Surveys Across the Tree of Life and Biomes

**DOI:** 10.1111/1755-0998.70088

**Published:** 2025-12-12

**Authors:** Henry F. N. Lankes, Lene Bruhn Pedersen, Rasmus Stenbak Larsen, Kathrin Rousk, Anders Priemé, N'golo A. Koné, Natasha de Vere, Jacob Heilmann‐Clausen, Michael Poulsen, Kristine Bohmann, Kasun H. Bodawatta

**Affiliations:** ^1^ Section for Molecular Ecology and Evolution, Globe Institute University of Copenhagen Copenhagen Denmark; ^2^ Section for Ecology and Evolution, Department of Biology University of Copenhagen Copenhagen Denmark; ^3^ Section for Terrestrial Ecology, Centre for Volatile Interactions, Department of Biology University of Copenhagen Copenhagen Denmark; ^4^ Section for Microbiology, Department of Biology University of Copenhagen Copenhagen Denmark; ^5^ Station de Recherche en Ecologie du Parc National de la Comoé Abidjan Cote d'Ivoire; ^6^ Unité de Formation et de Recherche en Sciences de la Nature (UFR‐SN) Université Nangui Abrogoua Abidjan Cote d'Ivoire; ^7^ Natural History Museum of Denmark University of Copenhagen Copenhagen Denmark; ^8^ Section for Biodiversity, Globe Institute University of Copenhagen Copenhagen Denmark

**Keywords:** biodiversity, bryophyte, environmental DNA, invertebrates, metabarcoding, microbes, temperate, tropical, vertebrates

## Abstract

Developments in the environmental DNA (eDNA) field have revolutionised our ability to map biodiversity by providing cost‐effective and non‐invasive means to survey organisms across the tree of life. In the terrestrial realm, a variety of eDNA sources have been employed, but we lack easily accessible and cosmopolitan sources of terrestrial eDNA. Here we document the value of a novel eDNA source for mapping lifeforms across the tree of life in temperate and tropical ecosystems: mosses (Bryophytes). First, we analysed eDNA from 25 moss patches collected using three sampling methods (swabbing, moss stubs, and washes of moss stubs) across three sites in Denmark. We detected 26 vertebrate species, 54 invertebrate genera, 21 vascular plant genera, and 553 bacterial and 210 fungal genera. Swabbing was sufficient to obtain eDNA, eliminating the need for destructive sampling of mosses. Subsequently, employing the swabbing approach in gallery forest and savanna ecosystems in Côte d'Ivoire we assessed its use for vertebrate detections. Metabarcoding of 29 moss swabs yielded 18 bird, 13 mammal, and two amphibian genera, confirming its applicability in the tropics. Our findings expand the current biodiversity monitoring toolkit by capitalising on a cosmopolitan and readily available terrestrial eDNA source.

## 1 Introduction

With the ever‐increasing global change and biodiversity crisis, we need effective and economical monitoring tools to assess biodiversity and ecosystem functioning under anthropogenic pressures and to evaluate conservation efforts. Environmental DNA (eDNA) analyses capitalise on a diversity of sources of DNA traces left behind by organisms suspended in water, present as airborne particles, or deposited in sediments, allowing detection of biodiversity by DNA sequencing (Bohmann et al. 2014). In terrestrial ecosystems, airborne particles have been used to detect land‐living lifeforms (Johnson and Barnes 2024), including microbes (Banchi et al. 2020; Serrano‐Silva and Calderon‐Ezquerro 2018), plants (Banchi et al. 2020), fungi (Abrego et al. 2024), insects, and vertebrates (Lynggaard et al. 2022; Roger et al. 2022). Active filtration of airborne particles is effective to characterise biodiversity (Bodawatta et al. 2025; Chen et al. 2018; Lynggaard et al. 2022; Polling et al. 2024; Roger et al. 2022), but the need for a power supply challenges its use in remote areas. This challenge is overcome with swabbing of surface‐accumulated eDNA on leaves (Lynggaard et al. 2023) and spider webs (Gregoric et al. 2022; Newton et al. 2024) to detect a range of taxa. However, leaves and spider webs are transient and depend on passive accumulation of eDNA. We therefore need alternative sources that naturally collect and store eDNA to estimate biodiversity across the tree of life in terrestrial ecosystems.

In marine environments, sponges (Porifera) are an accessible and natural eDNA sampler to survey biodiversity (Cai et al. 2022, 2024; Harper et al. 2023), as eDNA from a range of marine organisms, including fish, birds, and mammals, is trapped during filter feeding (Mariani et al. 2019). On land, mosses (Bryophytes) may serve as a comparable natural eDNA trap. Mosses are perennial and found from the subarctic to the tropics (Geffert et al. 2013), spanning a range of ecosystems, including forests, cities, and even aquatic (Rusin 2016) and desert (Scott 1982) habitats. Mosses have pseudo‐roots that are inefficient at absorbing nutrients; however, their dense growth forms and high surface area to leaf volume ratio (Rusin 2016) allow the capture of water and nutrients from the atmosphere (Glime 2007). This coincides with the accumulation of airborne particles that have been capitalised on for monitoring atmospheric pollution for decades (Świsłowski et al. 2022; Varela et al. 2023).

**FIGURE 1:**
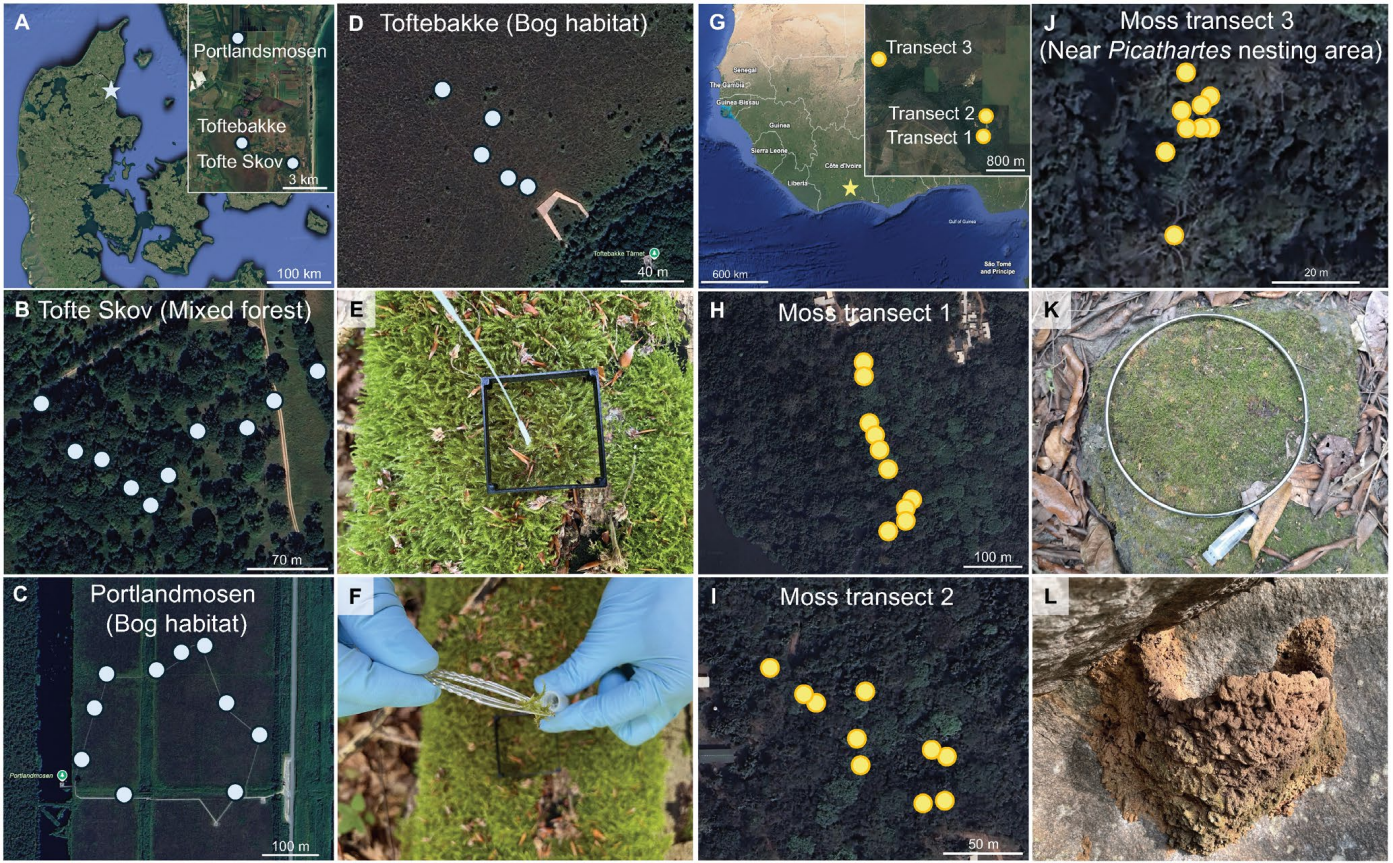
Sampling of moss for environmental DNA analyses. Sampling was carried out in Lille Vildmose Nature Area, Denmark (A–F) and Lamto Ecological Research Station, Côte d'Ivoire (G–L). The three sampling transects in Lille Vildmose were in bogs; Toftebakke (B) and Portlandmosen (C) and in forest; Tofte Skov (D) (Table S1). Samples were collected on 6th and 7th of September 2023. (E) Image illustrating the moss swabbing approach where a 6 × 6 cm square area of moss was sampled. (F) Image of the sampling of one to two moss stubs. (G) Map of West Africa indicating the location of Lamto Ecological Research Station in Côte d'Ivoire and three sampling transects. (H–J) Individual maps depicting the sampling points of each of the transects (transect 1 and 2 are in gallery forest and transect 3 in savanna). Samples were collected between 25th and 27th of March 2024. (K) Image showing the 15 cm diameter ring for sampling moss patches. (L) White‐necked rockfowl ( *Picathartes gymnocephalus* ) nest located near the third moss transect and used as a test for whether moss accumulated eDNA can store DNA traces from species after they have left an area (J).

Here, we test if mosses act as natural traps for eDNA in temperate and tropical ecosystems. First, to evaluate the possibility of surveying biodiversity, we characterised moss‐accumulated vertebrate (mammals and birds), invertebrate, vascular plant, bacterial, and fungal DNA using three sampling methods, at three sites within the Lille Vildmose Nature Area in Denmark (Figure 1A–F). After validating the approach, we adopted it to survey vertebrate communities in the tropics across three savannah and gallery forest sites at the Lamto Ecological Research Station in Côte d'Ivoire (Figure 1G–L). Our results demonstrate that mosses trap eDNA that can be used to survey terrestrial biodiversity across the tree of life in temperate and tropical biomes.

## 2 Materials and Methods

### 2.1 Field Sites and Sample Collection

We collected moss samples in Denmark and Côte d'Ivoire. In Denmark we collected moss samples in the Lille Vildmose Nature Area in two bogs: Portlandmosen and Toftebakke, and in a forest, Tofte Skov (Figure 1A–D, Table S1) in early autumn on 6–7th of September 2023. At Portlandmosen and Tofte Skov we sampled 10 moss patches in each, while at Toftebakke we sampled five moss patches (Figure 1). At each habitat we ensured sampling morphologically similar moss patches and we collected two different sample types per patch: moss swabs and 1–2 moss stubs (Figure 1E,F). We swabbed an area of 6 × 6 cm per moss patch with a sterile COPAN mini FLOQ swab for 3 min (Figure 1E). We swabbed the stems and leaves of the moss patch, while avoiding touching the pseudo‐roots and soil. For moss stubs we collected 1–2 moss stems with leaves from the middle of the swabbed area (prior to swabbing) with sterile forceps (Figure 1F). Both swabs and stubs were stored in 500 μL Longmire's buffer and kept at 4°C until DNA extractions. This resulted in a total of 25 swab and 25 stub samples.

After confirming the potential for using moss‐derived eDNA for biodiversity surveys in Denmark, we explored its use in the tropics. We used the sampling method found to perform the best in Denmark (see Section 3), moss swabbing (Figure 1E), to sample 29 moss patches across three sampling sites (Figure 1G–J; Table S1) at the Lamto Ecological Research Station in Côte d'Ivoire. Here we swabbed moss patches in gallery forest (transect 1: 10 moss patches and transect 2: 10 moss patches) and savanna (transect 3: nine moss patches) (Figure 1G–J). Samples were collected in the late dry season from 25th to 27th of March 2024 (Table S1). To increase the amount of eDNA captured, we increased the sampling area to 15 cm diameter (Figure 1K) while reducing the swabbing time to 2 min. Furthermore, based on the success of using sterile cotton swabs to sample leaf surface‐accumulated vertebrate eDNA in a tropical rainforest habitat in Uganda (Lynggaard et al. 2023), we used sterile cotton swabs (Heinz Herenz: HERE1030228) in Côte d'Ivoire. Swabs were stored as the Danish samples. One of the transects (transect 3; Figure 1J) was near a nesting area of *Picathartes gymnocephalus* (white‐necked rockfowl) and our sampling time was outside its breeding season. Sampling in this transect thus allowed us to evaluate if mosses can retain eDNA from organisms after they have left an area. Here, we also swabbed three old nests (Figure 1K) as positive controls.

### 2.2 DNA Extractions

We used a modified protocol of the Qiagen DNeasy Blood and Tissue kit (Hilden, Germany). DNA extractions were carried out in a pre‐PCR DNA extraction lab and done separately for swabs, stubs, and washes (Longmire's buffer from the moss stubs). For swabs, we first moved the swab using a flame‐sterilised metal forceps into a sterile 2 mL DNA LoBind tube along with 200 μL of Longmire's buffer. Subsequently, we added 225 μL of ATL buffer and 25 μL of proteinase K along with approximately 1 g of acid‐washed sterile glass beads (Sigma‐Aldrich: G8772‐100G). The mixture was vortexed for 5–10 s, followed by shaking in a TissueLyser II (Qiagen) for 30 s at 30 hertz. Samples were then incubated overnight at 56°C on a rotor. For the wash, we vortexed the full sample with moss stubs in Longmire's buffer for 10–15 s, followed by bead‐beating on a TissueLyser II for 5 min at 15 hertz. Then we transferred 200 μL of the wash to a new 2 mL DNA LoBind tube and added 225 μL of ATL buffer and 25 μL of proteinase K. Approximately 1 g of acid washed sterile glass beads were added to the mixture and vortexed for 10–15 s. Samples were then incubated as was done for the swab samples. For moss stubs, after removing 200 μL of the wash, we transferred appx. 25 ng of stubs using a flame‐sterilised metal forceps into a new 2 mL DNA LoBind tube. The samples were then treated as the moss swab samples. Subsequent steps of the DNA extraction protocol were the same for the three different sample types. Briefly, we added 250 μL AL buffer and 250 μL ethanol (96%–100%) to the digest and followed the original protocol of the extraction kit. During the elution step, after adding 45 μL of AE buffer to the spin columns, we incubated the samples for 5 min at 37°C. This step was repeated twice giving us a total elution volume of 90 μL. For Côte d'Ivoire samples, we followed the same moss swab extraction protocol. The extracted DNA was purified using the Zymo OneStep PCR Inhibitor Removal Kit (Zymo Research, USA). Purified DNA extracts were stored at −20°C. During extractions, we included various kinds of negative controls, including negative extraction controls, Longmire's buffer negative controls, and sterile swab negative controls. Additionally, we evaluated potential DNA contamination from the DNA extraction laboratory by extracting DNA from a tube of Longmire's buffer left open for 24 h in the laboratory prior to extractions.

### 2.3 Amplicon Sequencing

For amplicon sequencing of the 75 Danish moss samples, we used primer sets targeting six different taxonomic groups across the tree of life (Table S2). For macro‐organisms we used four primer sets: the BirT primer set targeting a 260 bp (excluding primers) mitochondrial 12S region for birds (Thalinger et al. 2023), the 16Smam primer set targeting a 90 bp (excluding primers) mitochondrial 16S region for mammals (Taylor 1996), the fwh primer set targeting a 205 bp (excluding primers) Cytochrome c oxidase 1 (CO1) region for invertebrates (Vamos et al. 2017), and the Trac01 primer set targeting a 267 bp (excluding primers) ITS1 region for vascular plants (Taberlet et al. 2018). Within these primer sets, primers were labelled by addition of 6‐7 nucleotides to the 5′ ends to create sets of unique primers, enabling the labelling of each sample's PCR replicates with a unique tag combinations during the metabarcoding PCRs (Bohmann et al. 2022). For PCR amplification of the Côte d'Ivoire samples we only used the BirT and 16Smam primer sets for macro‐organisms. Prior to metabarcoding, we conducted quantitative PCRs (qPCRs) on a dilution series (undiluted, 1:2, and 1:5 dilution) of DNA from a subset of the samples to identify the optimal template DNA amount to avoid PCR inhibition and to identify the number of PCR cycles needed in the metabarcoding PCR amplifications (Murray et al. 2015). Quantitative PCRs were conducted in 20 μL reactions containing 2 μL template, 1 unit AmpliTaq Gold^TM^ DNA polymerase, 1× AmpliTaq Gold^TM^ PCR buffer, 2.5 mM MgCl_2_ and 0.2 mM dNTPs (Applied Biosystems, Waltham, MA, USA), 0.5 mg/mL Recombinant Albumin (New England BioLabs), 0.6 μM forward and 0.6 μM reverse primer, 1 μL of SYBR green ROX (1:4 SYBR green (Invitrogen) to ROX reference dye (Invitrogen) with 200 parts high‐grade DMSO). For 16Smam primers we added 3 μM human blocker (Boessenkool et al. 2012). Reactions included 50 PCR cycles followed by a dissociation curve replacing the final elongation step (see Table S2 for PCR conditions). Undiluted negative controls were included and showed no signs of contamination.

Metabarcoding PCRs were conducted using the same PCR master mix, but without SYBR green ROX. PCR conditions, the number of cycles, and the dilution of template used for metabarcoding PCRs can be found in Table S2. We conducted four PCR replicates for each sample and PCR products of each set of replicates were pooled based on band intensity on a 2% agarose gel. Amplicon pools were purified using HighPrep PCR magnetic beads (MagBio Genomics, USA). Illumina sequencing adapters were then ligated using the PCR‐free TagSteady library building protocol (Caroe and Bohmann 2020). Libraries were then purified similar to amplicon pools and quantified using the NEBNext Library Quant Kit for Illumina (New England BioLabs, USA). Quantified libraries were then pooled equimolar before sequencing. Libraries from Denmark were sequenced on an Illumina MiSeq v3 (2 × 300 cycles) platform (Illumina, USA), while libraries from Côte d'Ivoire were sequenced on ca. 10% of an Illumina NovaSeq 6000 SP lane (XP, v1.5, 250 bp paired‐end) at the GeoGenetics Sequencing Core, University of Copenhagen, Denmark.

Two primer sets targeting bacteria and fungi respectively were used on all Danish samples. For these we used a two‐step PCR strategy to amplify metabarcoding markers and to build libraries (Bohmann et al. 2022). We used 341F and 806R primers targeting the V3–V4 region of the bacterial 16S rRNA gene (464 bp ‐ excluding primers) (Sundberg et al. 2013; Yu et al. 2005) and gITS7 and ITS4ngs primers targeting the ITS region of fungi (460 bp ‐ excluding primers) (Ihrmark et al. 2012; Tedersoo et al. 2015) (Table S2). Initial PCRs were conducted with un‐tagged primers with Illumina overhangs. These PCRs were conducted only once per sample DNA extract. The 25 μL reactions contained 5 μL of HiFi buffer (PCR Biosystems, UK), 0.25 μL of Polymerase HiFi (PCR Biosystems, UK), 1 μL of 10 μM forward and reverse primers, and 2 μL template DNA (see PCR conditions in Table S2). We only conducted one PCR replicate per sample as microbial DNA is not as scarce as macrobial DNA in environmental samples and as PCR replicates of microbial samples tend to yield compositionally similar communities (Reboleira et al. 2022). Following amplification, the PCR products were purified using HighPrep PCR magnetic beads (0.65:1 beads:PCR product) (MagBio Genomics, USA). In the following (second) PCR, Illumina sequencing adapters and sample‐specific dual indexes were added using PCRBIO HiFi (PCR Biosystems Ltd., UK) for 15 cycles. The resulting amplicon libraries were purified similarly to the first PCR products and the SequalPrep Normalisation Plate (96) Kit (Thermofisher, USA) was used to normalise the library concentrations. Subsequently, libraries were pooled upconcentrated using the DNA Clean and Concentrator‐5 Kit (Zymo Research, USA). The final 9pM sequencing pool was sequenced on an Illumina MiSeq v3 (2 × 300 cycles) platform (Illumina, USA) at the Section of Microbiology, University of Copenhagen.

### 2.4 Sequence Processing and Bioinformatics

For macro‐organisms, removal of Illumina adapters and low quality reads and merging of paired reads were conducted using AdapterRemoval 2.3.2 (Schubert et al. 2016), with the following parameters: minlength = 100, qualitybase = 33, minquality = 28. Subsequent sequence processing was done with the begum pipeline (Yang et al. 2021). First, the Begum.py_sort command was used for primer trimming and demultiplexing of the samples. The Begum.py_filter command was then used to filter sequences according to sequence copy number and across a sample's PCR replicates. For the vertebrate detections with the BirT and 16Smam primer sets, we did not apply filtering of sequences across the four PCR replicates, i.e., we retained all sequences found in all four PCR replicates. This was done as detections of vertebrates were stochastic across each sample's PCR replicates (c.f. (Alberdi et al. 2017)). For the BirT and 16Smam primer sets, minimum sequence thresholds within PCR replicates were set to five for samples from Denmark and 50 for samples from Côte d'Ivoire. In contrast, the fwh and Trac01 primer sets produced more robust detections with less stochasticity, which for the fwh primer set allowed us to only retain sequences found in at least three of each sample's four PCR replicates (with a minimum sequence copy number of five) and for the Trac01 primer set allowed us to only retain sequences found in at least two of each sample's four PCR replicates (with a minimum sequence copy number of five). Minimum sequence length was set to 250 bp for BirT, 90 bp for 16Smam, 180 bp for fwh, and 267 bp for Trac01. Using the convertToUSearch.py command we trimmed the sequences with lmin and lmax set to 260 and 300 bp for BirT, 90 and 110 bp for 16Smam, 175 and 205 bp for fwh, and 250 and 300 bp for Trac01. Filtered sequences were then clustered at 97% similarity using sumaclust‐1.0.31 (Mercier et al. 2013) and the operational taxonomic units (OTU) tables were created using tabulateSumaclust.py.

The OTU sequences were blasted against the NCBI database to assign taxonomy and OTUs with 99%–100% identity and query cover to a species were considered a match. OTUs with 98%–99% identity were assigned at the genus level, while sequences with 97%–98% identity were assigned at the family level. Subsequently we manually curated the OTU tables to create taxa tables. If an OTU was identified as a closely related species outside of Denmark, we considered this OTU to be from a species occurring in Denmark; if multiple species within a genus exist in Denmark, we assigned the OTU at the genus level. For example, we assigned *Matricaria aurea* *m* (a Mediterranean plant species) and *Eriophorum callitrix* (an Arctic plant species) to their respective genera *Matricaria* spp. and *Eriophorum* spp. as there are multiple other species within these genera in Denmark. In the Côte d'Ivoire 16Smam dataset, we detected *Capreolus capreolus* in two samples. Given that this genus does not occur in West Africa we removed it from the table, as it most likely represents a sequence mis‐annotation in the database. All OTUs identified to the same species were merged. Moreover, if multiple OTUs had a match for a single genus, and the sequences belonging to these OTUs were always found in the same samples, we merged the OTUs at the genus level. Taxa with identification outside the target taxonomic groups for a given primer set were removed from the taxa tables as were human and domesticated animals. From the plant taxon table, we removed any sequences assigned to non‐vascular plants. For subsequent analyses we only used taxa that were identified to at least the genus level.

Fungal and bacterial sequences were processed with the DADA2 pipeline (Callahan et al. 2016) within Qiime2 (Bolyen et al. 2019) with default parameters. Sequences were assigned to amplicon sequence variants (ASVs) at 100% similarity. Bacterial ASVs were assigned to taxonomy using the Silva 138.2 (Quast et al. 2013) reference database and fungal ASVs were assigned to taxonomy using the UNITE database (Nilsson et al. 2019). The prevalence method in the Decontam package (Davis et al. 2018) was used to identify and remove potential contaminant ASVs found in the negative controls (extraction, buffer and PCR controls) within the phyloseq package (McMurdie and Holmes 2013) in R 4.4.2 (R Core Team 2022). Finally, any sample with fewer than 3000 sequences was removed from the dataset.

### 2.5 Statistical Analyses

All analyses were conducted in R 4.4.2 (R Core Team 2022). BirT and 16Smam taxon tables were concatenated (using only presence/absence data) to generate a vertebrate taxon table. Observed taxonomic richness of vertebrates, invertebrates and plants was calculated individually for each of the higher taxonomic groups. We visualised the number of samples with different taxa belonging to different genera using alluvial plots in the ggalluvial package (Brunson and Read 2023).

Filtered bacterial and fungal datasets were analysed separately using the phyloseq package (McMurdie and Holmes 2013). ASVs shared between sample types and sampling sites were visualised through generating upset plots with the UpSetR package (Conway et al. 2017). We visualised the microbial community compositions through generating principal component analyses plots with Bray‐Curtis distances. Statistical significance of sample type and sampling area on microbial communities was evaluated using Permutational multivariate analyses of variance tests (PERMANOVAs) with the adonis function in the vegan package (Oksanen et al. 2022). PERMANOVAs were conducted with 10,000 permutations, with the by parameter set to “margin” and the strata parameter set to sample points. We used the microeco package (Liu et al. 2021) along with the ggnested package (Teunisse 2024) to generate bar charts and heat maps to visualise the relative abundance of bacterial and fungal taxa. Moreover, since bacteria detected in samples could either represent eDNA captured or moss‐associated taxa, we explored the ecology of the detected bacterial genera by investigating their known affiliations with moss, plants, and/or soils. We gathered this information by a search of each genus name in Google Scholar and the List of Prokaryotic names with Standing in Nomenclature (Parte et al. 2020) and by surveying papers reporting bacterial microbiomes of mosses (Baev et al. 2024; Holland‐Moritz et al. 2018; Ishak et al. 2024; Wang et al. 2024). All figures were generated with the ggplot2 package (Wickham 2016).

## 3 Results

### 3.1 Mosses Capture eDNA From Local Vertebrates, Invertebrates, Plants, and Microbes in Temperate Bog and Forest Habitats

**FIGURE 2:**
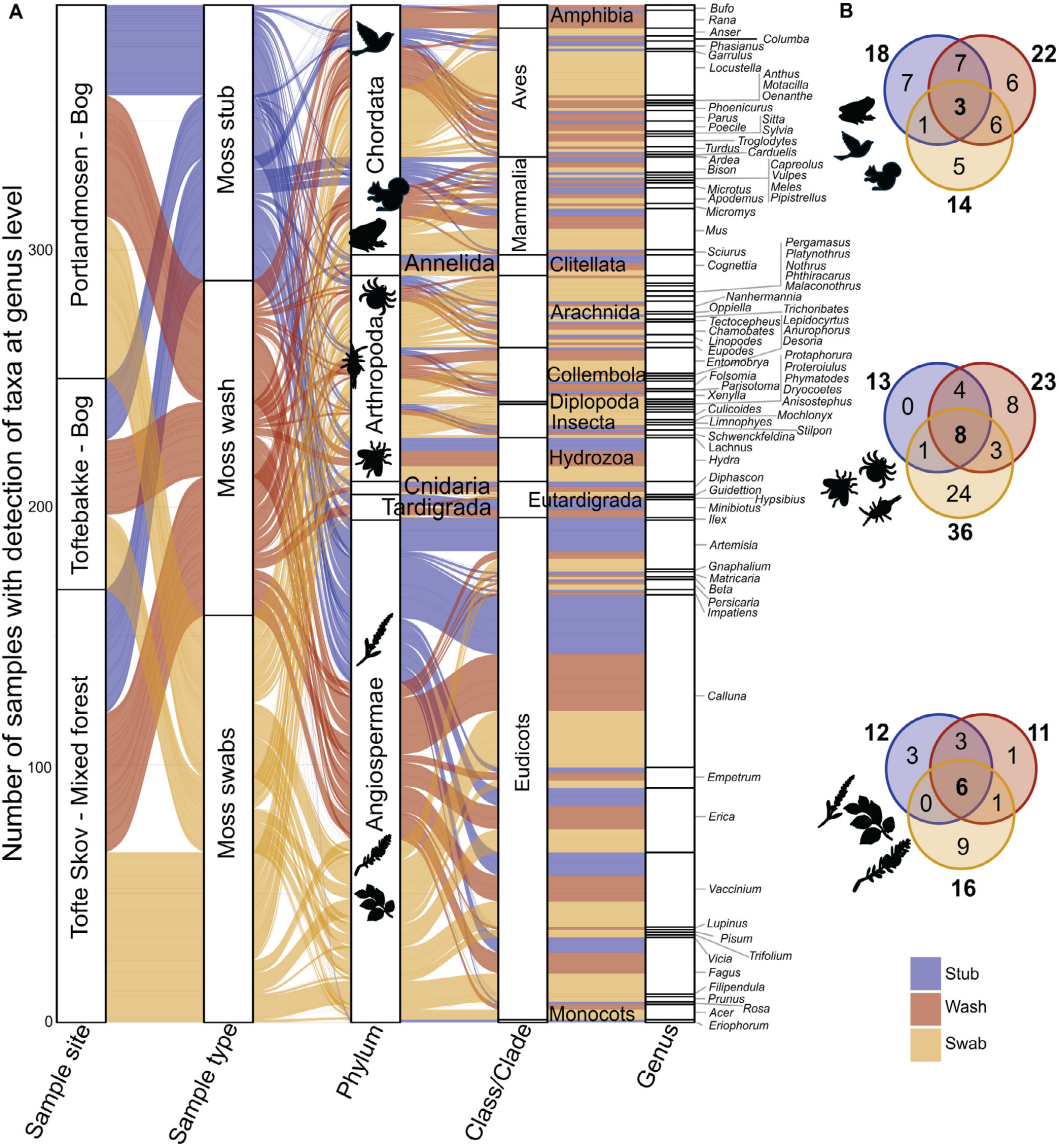
Vertebrates, invertebrates, and plant genera detected from DNA metabarcoding. (A) Alluvial plot of the frequency of genera detected in moss samples collected in three sampling sites in Lille Vildmose Nature Area and with the three moss sampling methods. In the genus column, the size of the boxes reflects the number of samples where each genus was detected. (B) Venn diagrams of the shared and unique number of taxa between the sampling approaches (represented by colours) for vertebrates (top), invertebrates (centre) and plants (bottom).

In temperate bog and forest habitats in Lille Vildmose in Denmark, we detected wild vertebrates in 49 of the 75 moss samples (swabs, moss stubs, washes of moss stubs) with an average of 2.06 ± 1.25 taxa per sample (average number of sequences ± SD: BirT: 41,273 ± 43,114; 16Smam: 21,056 ± 26,118). The 34 detected wild vertebrates spanned 18 bird, 14 mammal, and two amphibian taxa (Table S3, Table S4). Of the bird taxa, 13 were assigned to wild Danish species, including *Phoenicurus phoenicurus* (common redstart), *Sitta europaea* (Eurasian nuthatch), *Columba palumbus* (common wood pigeon), *Parus major* (great tit), and *Anser anser* (greylag goose) (Figure 2A; Table S5). Among the detected 14 mammal taxa, 11 could be identified at the species level, including *Bison bonasus* (European bison), *Meles meles* (European badger), and *Pipistrellus pygmaeus* (soprano pipistrelle). The two amphibian taxa were *Bufo bufo* (common toad) and *Rana arvalis* (moor frog) (Figure 2A; Table S5). All detected vertebrate species are known to live in Lille Vildmose. The majority of swab samples (22 of 25) yielded detections of at least one wild vertebrate taxon (1.9 ± 0.9), while only 14 washes (2.5 ± 1.6) and 13 stub samples (1.84 ± 1.28) yielded vertebrates (Table S5). Moss stub (18) and wash samples (22) yielded most vertebrate taxa, while only three taxa were detected with all three sampling methods (Figure 2B).

With the fwh primer set, we detected 48 invertebrate genera (2.02 ± 1.6 per sample) across 54 of 75 samples (average number of sequences ± SD: 6850 ± 11,323) (Table S6). Most taxa belonged to the phylum Arthropoda (40), followed by Tardigrada (5) and Annelida (2). Within the arthropods, the classes Arachnida (13 taxa), Collembola (16), and Insecta (9) dominated (Figure 2A; Table S5). All three moss sampling types yielded detections of eight invertebrate taxa at the genus level; however, swabs yielded both the highest number of (36) and most unique (24) taxa (Figure 2B; Table S5).

In the plant dataset, there were two moss taxa ( *Pleurozium schreberi* and *Hypnum* spp.) (Table S7), which are common in the sampling area in Lille Vildmose (Fritz 2014). However, we did not detect another common moss in the area ‐ *Sphagnum*. This could be caused by primer set specificity to vascular plants (Taberlet et al. 2018). For further analyses, we only considered vascular plant taxa (removing mosses). We detected 23 vascular plant taxa at the genus level (2.54 ± 1.14 per sample) across 73 of the 75 samples (average number of sequences ± SD: 13,110 ± 13,901) (Table S7). Most of the detected taxa were Eudicots, with only one monocot genus (*Eriophorum*—cottongrass) (Figure 2A; Table S4). Among the Eudicots, the orders Ericales (5 taxa), Rosales (4) and Fabales (4) dominated. Of the 25 taxa, 17 were identified to the species level and these included a diversity of plants found naturally in Lille Vildmose (e.g., common heather—*Calluna vulgaris*, crowberry— *Empetrum nigrum* , cross‐leaved heath— *Erica tetralix* , and sweet cherry— *Prunus avium* ). There were also domestic species, such as beet ( *Beta vulgaris* ) and pea ( *Pisum sativum* ). The three moss sampling types yielded six plant taxa, while swab samples detected the most taxa (16) (Figure 2B; Table S4).

**FIGURE 3:**
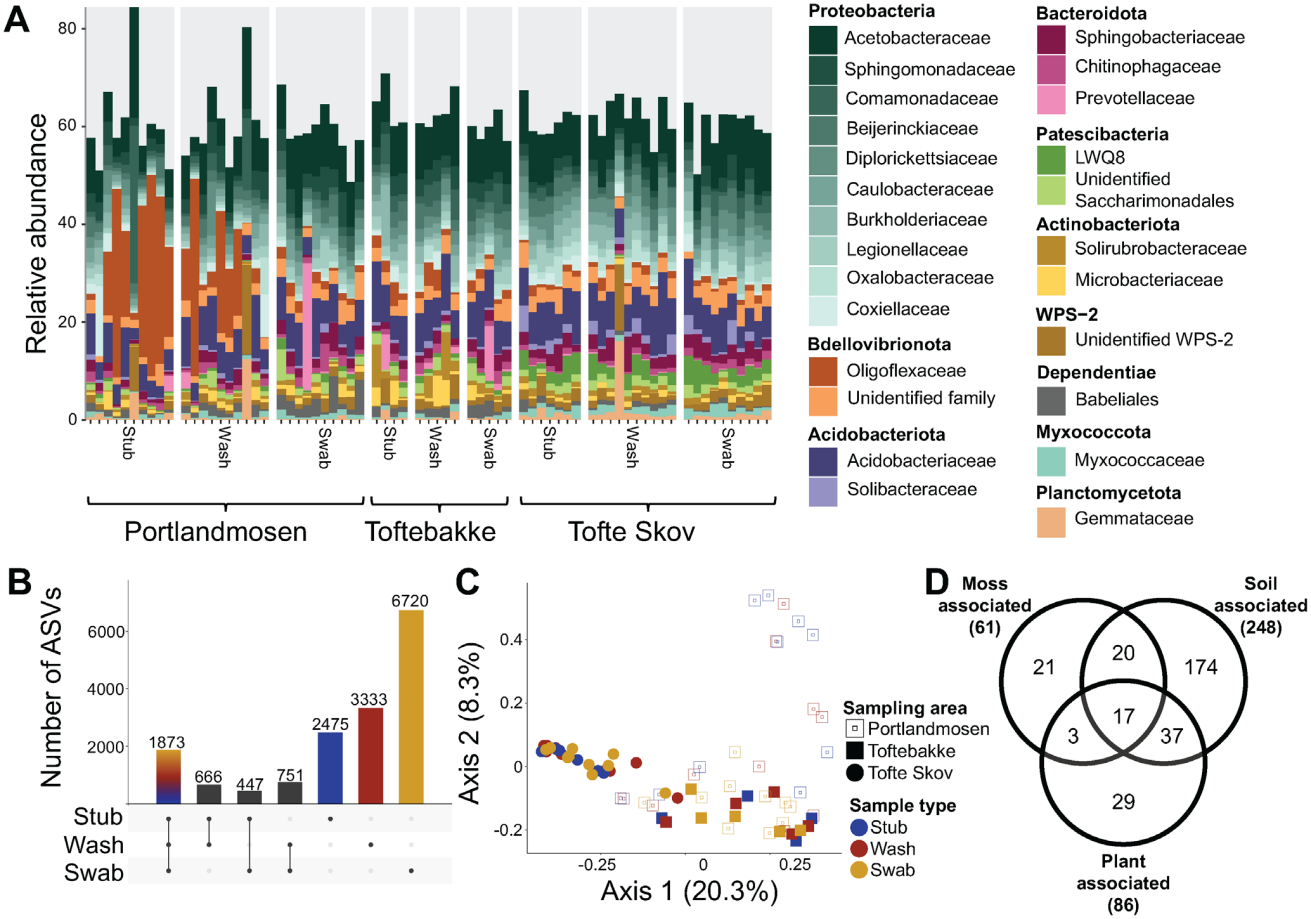
Diverse and site‐specific bacterial communities are detected with moss‐accumulated eDNA. (A) Relative abundances (%) of the top 20 bacterial families, stacked by phylum. (B) Upset plot showing the bacterial ASV sharing between sample types. (C) Ordination plot (PCoA) showing the bacterial community similarity between sample types and sites. Portlandmosen and Toftebakke are bog habitats, while Tofte Skov is a forest. (D) Venn diagram showing the association of detected bacterial genera with mosses, vascular plants, and soil based on our online searches (see Section 2).

After quality filtering and removal of potential contaminant ASVs (amplicon sequence variants), we retained 71 samples with bacterial sequences (average number of sequences ± SD: 23,871 ± 11,298). These sequences belonged to 16,288 ASVs that were assigned to 30 bacterial phyla. Proteobacteria accounted for most sequences across samples (average ± SD: 46.8% ± 5.7%), followed by Bdellovibrionota (9.5 ± 9.6), Acidobacteriota (9.5 ± 3.2), and Actinobacteriota (7.5 ± 3.8) (Figure 3A, Table S8). Of the identified ASVs, 1873 were shared between sample types, with swabs detecting most unique bacterial ASVs (6720) (Figure 3B). Bacterial community compositions differed significantly between sample types (PERMANOVA: F_2_ = 1.445, *R* ^2^ = 0.0345, *p* < 0.0001) and sample sites (PERMANOVA: F_2_ = 7.832, *R* ^2^ = 0.1851, *p* < 0.0001). Pair‐wise comparisons revealed that bacterial communities did not differ between stubs and washes (pairwise PERMANOVA: F_1_ = 0.5357, *R* ^2^ = 0.0121, *p*_adj_ = 1.000) or washes and swabs (F_1_ = 1.309, *R* ^2^ = 0.0266, *p*_adj_ = 1.000), but were marginally non‐significant between stubs and swabs (*F*_1_ = 1.849, *R* ^2^ = 0.0403, *p*_adj_ = 0.0603). Bacterial communities from the forest (Tofte Skov) differed significantly from the bogs Portlandmosen (F_1_ = 11.22, *R* ^2^ = 0.1694, *p*_adj_ = 0.0003) and Toftebakke (F_1_ = 9.335, *R* ^2^ = 0.1931, *p*_adj_ = 0.0003). The two bog sites also significantly differed from each other (Toftebakke vs. Portlandmosen: F_1_ = 2.261, *R* ^2^ = 0.0511, *p*_adj_ = 0.0123), even if the ordination analysis indicated that they were more similar (Figure 3C).

Through our online search, we identified 301 of 553 bacterial genera to be associated with moss, plants, and/or soils. Our knowledge of the habitats of environmental bacteria is generally limited and many genera only have a single described species. Thus, unknown species of these genera—and of more well‐described genera—may be found in other habitats than the type species. Only one genus (*Bryocella*) was identified as strictly moss‐associated (Dedysh et al. 2012). Sixty‐one genera were known to associate with mosses, 81 genera were common associates of plants, 248 genera could be categorised as soil associated, and 17 genera were common to moss, plants, and soil (Figure 3E; Table S9). This indicates that we capture ecologically diverse communities of bacteria, providing a snapshot of bacterial diversity of the ecosystems but not only moss‐associated bacteria.

**FIGURE 4:**
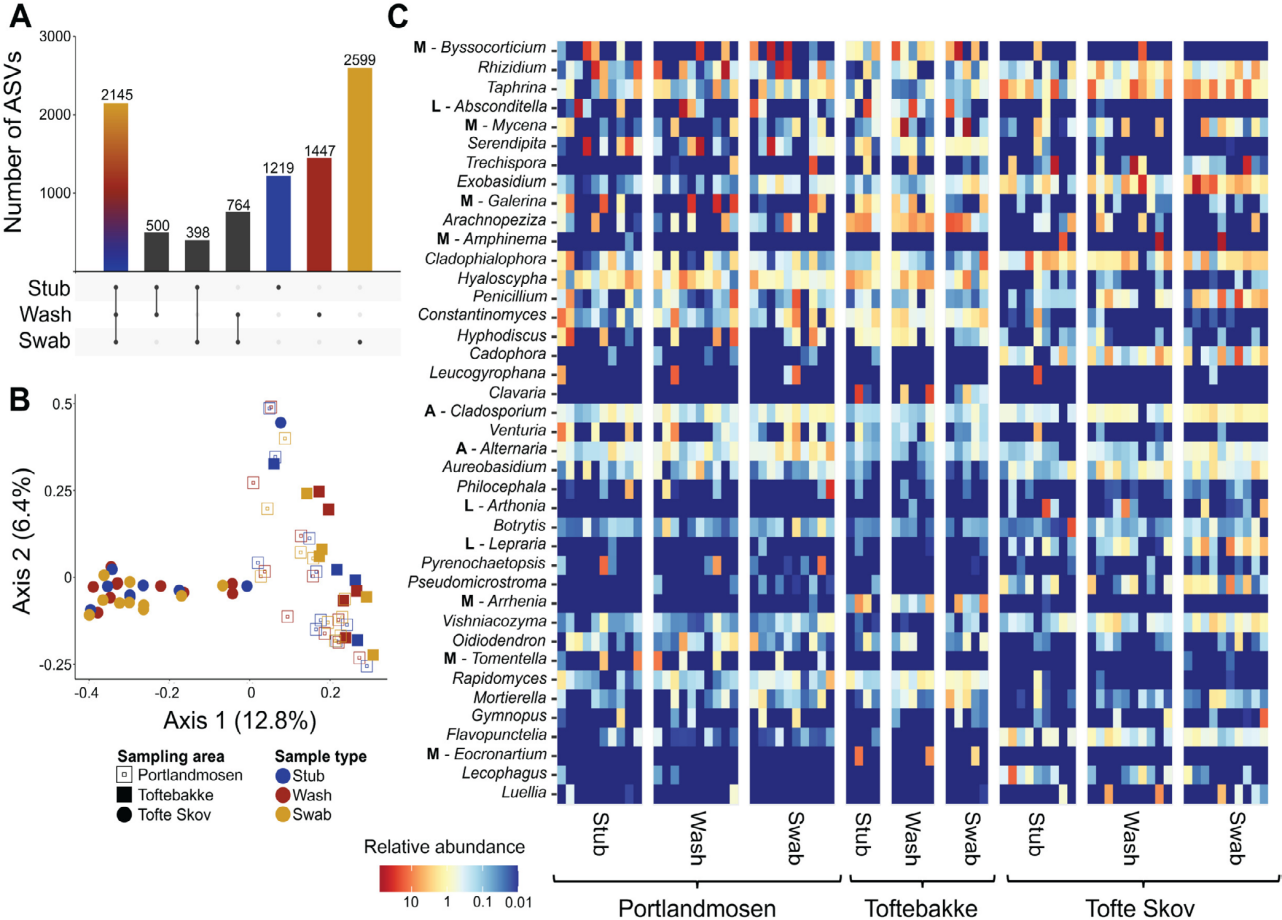
Moss‐accumulated eDNA capture a diversity of fungal taxa. (A) Upset plot showing the bacterial ASV sharing between sample types. (B) Ordination plot (PCoA) showing bacterial community similarity between sample types for Portlandmosen and Toftebakke (bog habitats) and Tofte Skov (forest). (C) Heat map of the relative abundance (%) of the 40 most abundant fungal genera detected across sample types and sampling areas. Bold upper‐case letters provide ecological insights of certain fungal genera (A, allergens; L, lichens; M, moss‐associated genera).

We obtained a total of 2,047,465 fungal sequences (average number of sequences per sample ± SD: 69,143 ± 18,368), which could be assigned to 9072 ASVs from 14 phyla from 73 samples. The majority of the ASVs belonged to the Ascomycota (40.5%), followed by Basidiomycota (25.8%) and Mortierellomycota (5.6%) (Table S10). The three moss sample types shared 2145 ASVs and swabs yielded most unique ASVs (Figure 4A). Fungal community compositions did not differ between sample types (PERMANOVA: F_2_ = 0.7517, *R* ^2^ = 0.0186, *p* = 0.9505) but did differ between sites (PERMANOVA: F_2_ = 5.768, *R* ^2^ = 0.1424, *p* < 0.0001). Pairwise comparisons revealed that fungal community composition differed among all sampling sites (pairwise PERMANOVA: Tofte Skov vs. Portlandmosen: F_1_ = 7.182, *R* ^2^ = 0.1119, *p*_adj_ = 0.0003; Tofte Skov vs. Toftebakke: F_1_ = 7.267, *R* ^2^ = 0.1506, *p*_adj_ = 0.0003; Portlandmosen vs. Toftebakke: F_1_ = 2.697, *R* ^2^ = 0.0603, *p*_adj_ = 0.0003).

Highly sporulating, ubiquitous microfungi, including *Aureobasidium, Penicillium* and the two well‐known and widespread allergens *Alternaria* and *Cladosporium* were among the most common genera (Figure 4C). This suggests that mosses possibly hold an imprint of regional fungal spore communities. Aligning with this, we detected several locally‐abundant lichen genera, such as *Absconditella*, *Arthonia*, and *Lepraria* and the plant parasitic genus *Exobasidium*, which is specific to members of the heather family (Ericaceae) (Nannfeldt 1981). Moreover, we frequently detected moss‐associated macrofungal taxa, such as *Arrhenia*, *Eocronartium*, *Galerina*, and *Mycena* (Davey et al. 2013; Gulden et al. 2021) along with ectomycorrhizal genera, including *Amphinema, Byssocorticium*, and *Tomentella* that produce sporocarps in the forest floor, including on the underside of moss cushions (Anderson et al. 2013). This implies that eDNA from mosses also yields substrate‐relevant fungi and lichens. The detection of the highly cryptic lichen species *Absconditiella sphagnorum*, which is associated with sphagnum mosses, is noteworthy, as it has only been detected by thalli from two Danish localities, including the nearby Tofte Mose (Frøslev et al. 2025). Equally remarkable is the widespread detection of the sphagnum‐associated ascomycete *Arachnopeziza japonica*, which was recently reported as new to Europe (Kosonen et al. 2021), but so far has remained undetected from Denmark based on presence of sporocarps. For many other fungal groups, species detection was limited, exemplified by the detection of only three of the ten most abundant wood‐inhabiting fungi known from the area (Tøttrup et al. 2013).

### 3.2 eDNA From Moss Swabs Capture Local Vertebrate Communities in the Tropics

**FIGURE 5:**
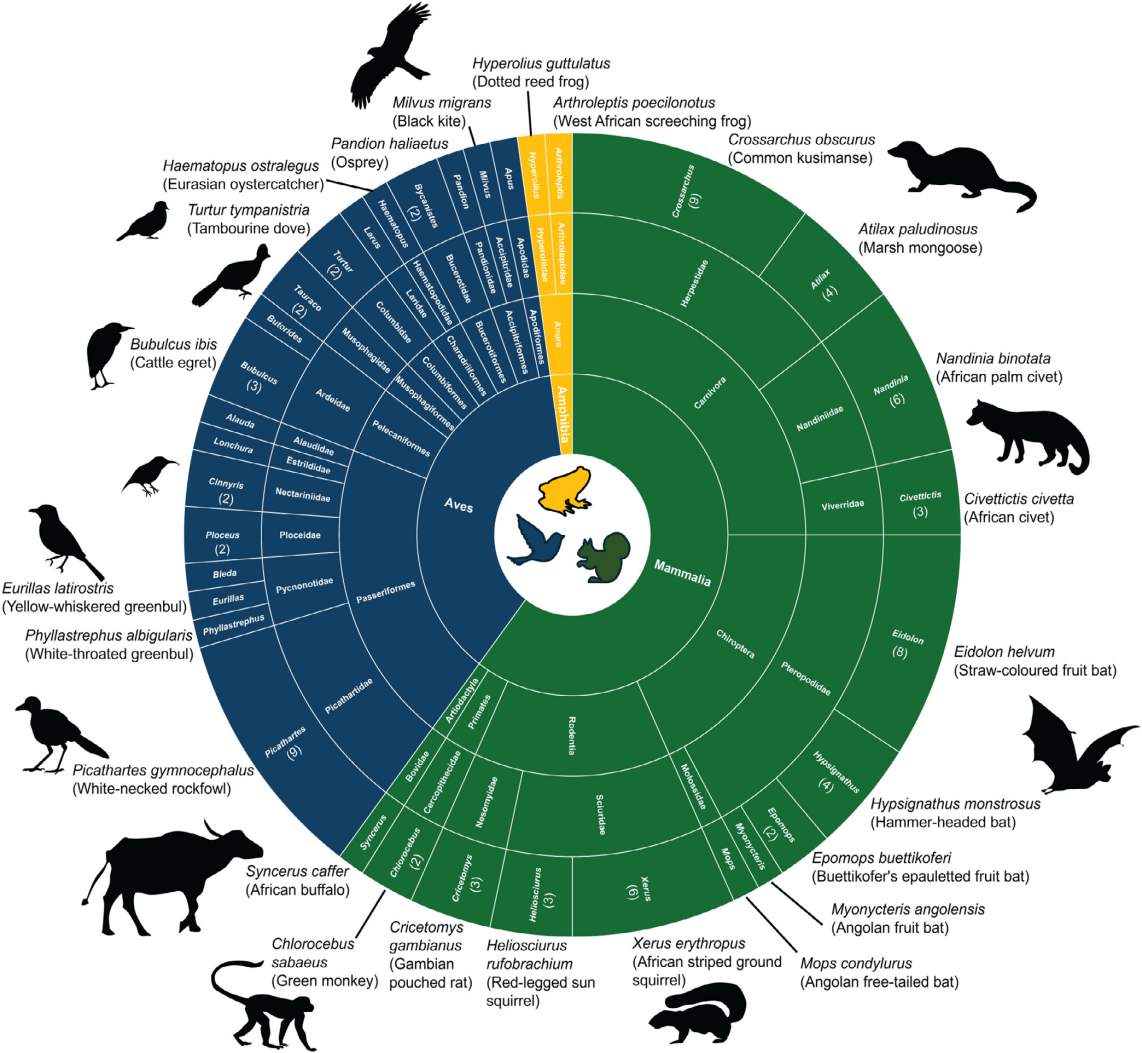
Vertebrate taxa detected by metabarcoding of moss swabs collected at forest and savanna sites at the Lamto Ecological Research Station in the Côte d'Ivoire. Crona chart of the proportions (based on the frequency of each taxon found across samples) of vertebrate taxa identified at the genus or species level. The number of samples where each taxon was detected is listed in parenthesis in the outer circle (taxa without numbers were only detected once). Illustrations depict representative taxa (species or genera) for the most common families.

After removal of domesticated taxa and taxa that could not be identified at the genus level, 19 swab samples from the BirT dataset (average sequences per sample ± SD: 164,999 ± 191,097) and 24 swab samples from the 16Smam dataset yielded detections (average number of sequences ± SD: 252,002 ± 255,431) (Tables S11 and S12). Overall, we detected 37 vertebrate taxa in 27 of the 29 collected swab samples, representing 19 birds, 16 mammals, and two amphibian taxa (3.4 ± 1.9 per sample). The 19 bird taxa represented a diversity of bird orders including Passeriformes (8 taxa), Columbiformes (2), Accipitriformes (2), Pelecaniformes (2), and Bucerotiformes (1) (Figure 5). Eight of these 19 taxa could be assigned to a species (Figure 5), and these included locally occurring taxa such as white‐necked rockfowl ( *Picathartes gymnocephalus* ), tambourine dove ( *Turtur tympanistria* ), black kite ( *Milvus migrans* ), and yellow‐whiskered greenbul ( *Eurillas latirostris* ). Despite sampling outside the breeding season of *P. gymnocephalus* , all sampled moss patches near the *P. gymnocephalus* nesting area (Figure 1J,L) contained DNA from the species (Figure 5; Table S5). All detected bird genera were expected from the study area at the time of sampling, except for the Eurasian oystercatcher ( *Haematopus ostralegus* ), but this species is found in the wider region during the northern winter. The 16 mammal taxa represented five orders: Chiroptera (6 taxa), Carnivora (4), Rodantia (4), Primates (1), and Artiodactyla (1) (Figure 5). These taxa include common wild mammals, such as the common kusimanse ( *Crossarchus obscurus* ), green monkey ( *Chlorocebus sabaeus* ), marsh mongoose ( *Atilax paludinosus* ), straw‐coloured fruit bat ( *Eidolon helvum* ), hammer‐headed bat ( *Hypsignathus monstrosus* ), and African striped ground squirrel ( *Xerus erythropus* ) (Figure 5). All detected mammals are known from the study area (Abbadie et al. 2006; Barbault 1976; Thiollay 1998). The two detected amphibian species were West African screeching frog ( *Arthroleptis poecilonotus* ) and the dotted reed frog ( *Hyperolius guttulatus* ) (Figure 5), both of which are known in the area.

## 4 Discussion

Our findings confirm that moss‐derived eDNA can be used to detect biodiversity across the tree of life in temperate ecosystems and for vertebrates in tropical ecosystems. We captured taxonomically diverse locally occurring taxa in Denmark and Côte d'Ivoire (Figures 2 and 5), with clear indication of fine‐scale spatial resolution of communities across sampled transects, especially for microbes (Figures 3 and 4, Table S5). However, we only captured a small proportion of the known vertebrate and invertebrate diversity of the sites, and there was high stochasticity in detections across samples. These trends align with early stages of exploring other eDNA sources, such as water (Takahashi et al. 2023), soil (Andersen et al. 2012), sponges (Mariani et al. 2019), air (Lynggaard et al. 2022), leaf swabs (Lynggaard et al. 2023), and invertebrate‐derived eDNA (Schnell et al. 2012), where methodological optimisations have improved the robustness of taxonomic detections (c.f. (Bodawatta et al. 2025; Takahashi et al. 2023)). Given that our aim was to evaluate if moss‐derived eDNA could satisfactorily detect representative—not necessarily complete—biodiversity, our findings provide the first empirical evidence in favour of this role.

We detected vertebrates varying in body size from large herbivores, such as European bison and African buffalo, to medium‐bodied animals, such as European badger, African civet, greylag goose, and hornbills, and small‐bodied rodents and birds such as *Apodemus* mice, the African striped ground squirrel, the yellow‐whiskered greenbul, and grasshopper warblers (Table S5). Capturing flying vertebrates that are unlikely to be in direct contact with mosses supports that mosses trap airborne eDNA or DNA from fecal droppings. The detection of white‐necked rockfowl near nesting sites outside the breeding season also implies that moss stores eDNA long after the focal organisms have left. Unsurprisingly, we detected taxa known to associate with moss, such as five tardigrade genera (Gasiorek et al. 2024), six fungal genera (Anderson et al. 2013; Davey et al. 2013; Gulden et al. 2021), and many bacteria (Ishak et al. 2024). Site specificity in bacterial and fungal communities suggests that moss samples can be representative of environmental microbial profiles for an area and/or moss host. Although our sampling methods (swabs vs. moss stubs) differed for survey outcomes, methodological optimisations will allow mosses to become a valuable eDNA source for terrestrial biodiversity monitoring.

With the exception of vertebrates, the swabbing approach yielded the highest diversity for all taxonomic groups. This is encouraging, as it provides the opportunity to sample in a way that is not destructive to the moss, i.e., without collecting moss stubs. Still, swabbing may miss certain taxa, as we, for example, only detected common toad and moor frog from stubs and washes (Table S5). Moss may accumulate amphibian DNA via direct contact, and the swab type (mini flocked swabs) used in Denmark may have influenced the retrieval of amphibian DNA. This inference is supported by the detection of amphibian taxa in Côte d'Ivore where we used cotton swabs. Future work should compare swab types (e.g., cotton, polyester, and foam) to identify the optimal types for eDNA collection. If disruptive sampling (e.g., the collection of moss) cannot be avoided, collecting more stubs may improve detections, even if this would also come with additional costs during DNA extraction due to the need for larger amounts of reagents. A better solution may be to increase the swabbed area or the swabbing depth of the moss patch. Our results indicate that this may be a way forward, as we detected more vertebrates after increasing the sampling area in Côte d'Ivoire compared to Denmark; however, this would need more rigorous testing as it could merely be due to the higher diversity in the tropics. Another avenue to increase detected diversity is through alternative storage methods such as freezing of the swabs or storing them in ATL buffer instead of Longmire's buffer, which preserves eDNA in water samples well (Spens et al. 2017) but was recently found to be suboptimal for airborne eDNA storage (Bodawatta et al. 2025). Finally, we sampled morphologically similar moss patches, but future work should integrate comparison of moss species to capture how differences in density, structure, and growth form may impact eDNA accumulation and storage.

The detection of white‐necked rockfowl near nesting sites after their breeding season was intriguing, as this points to the possibility of mosses storing eDNA after the focal taxa have left an area. This could make moss‐accumulated eDNA applicable to temporal conservation practices, such as in the search for rare species, detecting traces of invasive species, and assessing effects of conservation strategies over time (e.g., herbivore exclusion initiatives). However, all of these applications depend on the duration of time that mosses store eDNA. For example, if they store eDNA for longer (e.g., across seasons), then we would not be able to utilise moss‐accumulated eDNA to survey short‐term fluctuations in biodiversity, but rather to characterise total biodiversity in an area over time. However, if eDNA is stored for only 1–2 months, then moss‐accumulated eDNA can be useful to survey temporal changes in biodiversity, with the added benefit of detecting species that passed by the moss patch prior to sampling. In line with this, it will be important to assess moss eDNA capture across seasons to understand the temporal component of eDNA deposition.

The vertebrate diversity captured, particularly at Tofte Skov in Lille Vildmose, was less than when using active filtration of airborne eDNA, which was conducted during the time of moss sampling (Bodawatta et al. 2025). However, with active filtration of air in Tofte Skov, birds are overrepresented among the detected genera (34 bird genera) compared to mammals and amphibians (13 genera) (Bodawatta et al. 2025). Through moss‐acumulated eDNA, we detected a similar number of mammal and amphibian genera (10) but fewer birds (13 genera) than active filtration of air. Moss may thus be better for detecting ground‐dwelling vertebrates (i.e., mammals), which would complement approaches that actively filter air to capture DNA from flying vertebrates. We observed a similar trend of stronger representation of non‐bird vertebrates in moss swabs from Côte d'Ivoire (Figure 5). This trend was also apparent in our invertebrate dataset. While previous studies on airborne (Roger et al. 2022) and spider web (Gregoric et al. 2022) eDNA detected invertebrate communities containing more flying insects, moss sampling detected more ground‐dwelling and non‐flying invertebrates. These included springtails (Collembola), and multiple orders of mites (Oribatida, Sarcoptiformes, Mesostigmata), that are known to associate with mosses. Environmental DNA from moss may thus allow the capture of relatively more ground‐dwelling taxa in an ecosystem, particularly when sampling mosses growing at ground level. An evaluation of mosses growing in different forest strata could generate new insights to whether mosses accumulate eDNA from strata‐specific communities.

Our moss swab samples performed comparably to the limited number of studies from other surface‐accumulated eDNA sources, such as leaf swabs and spider webs. In a tropical Ugandan rainforest habitat, 24 leaf swab samples captured 52 wild vertebrate genera (Lynggaard et al. 2023), while our 29 moss swab samples from Côte d'Ivoire captured 32 vertebrate genera. These differences may result from factors related to sampling method (many leaves vs. one moss patch) and the vegetation and habitat type (e.g., savanna in Côte d'Ivoire vs. rainforest in Uganda (Pinkert et al. 2020)) along with biodiversity differences of the areas (Jenkins et al. 2013). Similarly, spider webs (*n* = 24) collected in a nature reserve in Southern Australia yielded 25 wild vertebrate species (Newton et al. 2024), as in our Côte d'Ivoire moss swab samples. However, a proper comparison between moss‐accumulated eDNA and other surface‐accumulated eDNA sources is needed to thoroughly evaluate the strengths and weaknesses of these respective sources.

Moss‐derived eDNA captured a diversity of plant, fungal, and bacterial taxa, which aligns with findings from previous eDNA‐based approaches (Abrego et al. 2024; Banerjee et al. 2022; Egan et al. 2014). The habitat‐specific microbial communities that represent a diversity of microbial ecologies imply that a simple moss swab can be used to survey microbial communities of a habitat. This removes the need for the collection of soil and the potential need to use soil‐specific extraction kits, making it a less labour‐intensive and more cost‐effective method. Similar to fungal communities characterised with airborne eDNA, moss‐derived eDNA is limited in detecting macrofungal taxa (Runnel et al. 2024). In our plant dataset, we detected mostly eudicots, while only detecting one species belonging to monocots (Figure 2), despite their abundance in the habitat. This might be a result of the specificity of the marker targeted, which is able to detect monocots (Storm et al. 2024), but seemingly suboptimally. Thus, the use of other markers targeting different genomic regions (e.g., rbcL and ITS2) might allow for the detection of more diverse plant communities (Jones et al. 2021). Our detection of fewer plant taxa may also be due to a lack of pollen captured in moss patches, or a suboptimal extraction method for retrieving plant DNA. Sampling took place in late September when less airborne pollen is deposited (Brennan et al. 2019), especially for the grass family (Poaceae) that typically flowers much earlier in the year. However, with further optimization and comparative studies, the method will be a promising addition to our current set of biodiversity monitoring tools.

## 5 Conclusions

Within this metabarcoding era of biodiversity monitoring, capitalising on the physiology of mosses, we demonstrate their use as a novel and readily available eDNA source in terrestrial habitats that can be used to survey biodiversity across the tree of life in both temperate and tropical ecosystems. The approach may also be very valuable in subpolar and polar regions, where moss can be the only available plant group. With future optimisations in sample collection and storage methods, swabbing a single moss patch may allow insights into the animals, plants, and microbes that inhabit or visit an ecosystem, adding a new eDNA source to the toolbox for eDNA‐based biodiversity monitoring.

## Author Contributions

K.H.B. designed the project, collected samples and analysed data; H.F.N.L. and L.B.P. conducted laboratory work and analysed data; R.S.L., N.A.K., and M.P. collected samples; K.R., A.P., N.V., J.H.‐C., and K.B. contributed to the analyses and interpretation of data. All authors contributed to writing and editing the draft.

## Funding

This work was supported by The Villum Foundation, Villum Experiment grant (57924) to Kasun H. Bodawatta. Kristine Bohmann was supported by a Carlsberg Foundation Semper Ardens: Accelarate grant (DF21‐0411), and Kathrin Rousk and Anders Priemé were supported by the Danish National Research Foundation (Danmarks Grundforskningsfond) within the Centre for Volatile Interaction (DNRF168).

## Disclosure

Benefit‐Sharing Statement: A research collaboration was developed with N.A.K. from the Côte d'Ivoire to collect moss swab samples. N.A.K. is included as a co‐author.

## Conflicts of Interest

The authors declare no conflicts of interest.

## Supporting information


**Table S1:** Metadata information of the sampling sites within sampling transects of Lille Vildmose, Denmark and the Lamto Ecological Research Station, Côte d'Ivoire. Table is in a separate excel sheet.

**Table S2:** Primer sets used for metabarcoding of focal taxonomic groups and their respective PCR parameters for metabarcoding PCRs.

**Table S3:** OTU table (Tab 1), full taxa table (Tab 2) and genus‐level taxa table (Tab 3) of BirT primer set for Lille Vildmose, Denmark. Table is in a separate excel sheet.

**Table S4:** OTU table (Tab 1), full taxa table (Tab 2) and genus‐level taxa table (Tab 3) of 16Smam primer set for Lille Vildmose, Denmark. Table is in a separate excel sheet.

**Table S5:** Proportion of samples with genus‐level taxonomic detections at the three sampling sites in Denmark (Tab 1) and Côte d'Ivoire (Tab 2). Table is in a separate excel sheet.

**Table S6:** OTU table (Tab 1), full taxa table (Tab 2) and genus‐level taxa table (Tab 3) of fwh (arthropods) primer set for Lille Vildmose, Denmark. Table is in a separate excel sheet.

**Table S7:** OTU table (Tab 1), full taxa table (Tab 2) and genus‐level taxa table (Tab 3) of Trac01 (vascular plant) primer set for Lille Vildmose, Denmark. Table is in a separate excel sheet.

**Table S8:** Cleaned bacterial ASV table of moss samples from Lille Vildmose, Denmark. Table is in a separate excel sheet.

**Table S9:** Ecological characterisation of bacterial genera found in moss samples of Lille Vildmose, Denmark. These categorisations were based on online search of each bacterial genus. Table is in a separate excel sheet.

**Table S10:** Cleaned fungal ASV table of moss samples from Lille Vildmose, Denmark. Table is in a separate excel sheet.

**Table S11:** OTU table (Table 1), full taxa table (Table 2) and genus‐level taxa table (Table 3) of BirT primer set for Lamto Ecological Research Station, Côte d'Ivoire. Table is in a separate excel sheet.

**Table S12:** OTU table (Table 1), full taxa table (Table 2) and genus‐level taxa table (Table 3) of 16Smam primer set for Lamto Ecological Research Station, Côte d'Ivoire. Table is in a separate excel sheet.

## Data Availability

OTU tables are submitted to the GBIF metabarcoding database (Denmark: Birds (https://doi.org/10.21373/dnkx4x), Mammals (https://doi.org/10.21373/k824zc), Invertebrates (https://doi.org/10.21373/43gmku), Vascular plants (https://doi.org/10.21373/hjp4dp); Ivory Coast: Birds (DOI: 10.21373/x2mgme), and Mammals (https://doi.org/10.21373/fnn9xq)). Raw sequences (multiplexed) of macro‐organisms are submitted to the Zenodo data repository (https://doi.org/10.5281/zenodo.15792957) and raw sequences of microbial sequences are submitted to the NCBI SRA database (Bioprojects: PRJNA1283502 (Bacteria) and PRJNA1283573 (Fungi)).
