# Supplementary material for: Moss‐Accumulated eDNA Is a Promising Source for Terrestrial Biodiversity Surveys Across the Tree of Life and Biomes
Source: Mol Ecol Resour. 2025 Dec 12;26(1):e70088. doi: 10.1111/1755-0998.70088 (PMC12701329; doi:10.1111/1755-0998.70088)
Supplement: Supplementary file 2 — Table S2: Primer sets used for metabarcoding of focal taxonomic groups and their respective PCR parameters for metabarcoding PCRs. [file MEN-26-e70088-s008.docx]

**Supplementary information**

**Moss as a novel and natural eDNA trap for terrestrial biodiversity monitoring across the tree of life and biomes**

Henry F. N. Lankes^1^. Lene Bruhn Pedersen^1^. Rasmus Stenbak Larsen^2^. Kathrin Rousk^3^. Anders Priemé^4^. N'golo A Koné^5^. Natasha de Vere^7^. Jacob Heilmann-Clausen^8^. Michael Poulsen^2^. Kristine Bohmann^1^. Kasun H. Bodawatta^1*^

^1^Section for Molecular Ecology and Evolution. Globe Institute. University of Copenhagen. Copenhagen. Denmark

^2^Section for Ecology and Evolution. Department of Biology. University of Copenhagen. Copenhagen. Denmark

^3^Section for Terrestrial Ecology. Department of Biology. University of Copenhagen. Copenhagen. Denmark

^4^Section for Microbiology. Department of Biology. University of Copenhagen. Copenhagen. Denmark

^5^Station de Recherche en Ecologie du Parc National de la Comoé. Abidjan. Cote d’Ivoire

^6^Unité de Formation et de Recherche en Sciences de la Nature (UFR-SN). Université Nangui Abrogoua. Cote d’Ivoire

^7^Natural History Museum of Denmark. University of Copenhagen. Copenhagen. Denmark

^8^Section for Biodiversity. Globe Institute. University of Copenhagen. Copenhagen. Denmark

**Supplementary tables**

Table S1: Metadata information of the sampling sites within sampling transects of Lille Vildmose, Denmark and the Lamto Ecological Research Station, Ivory Coast. Table is in a separate excel sheet.

Table S2. Primer sets used for metabarcoding of focal taxonomic groups and their respective PCR parameters for metabarcoding PCRs.

| Primer set | Target taxonomic group and amplicon size | Positive control species | DNA template | PCR conditions | | |
| --- | --- | --- | --- | --- | --- | --- |
|  |  |  |  | Cycles | Temp | Time |
| BirT (Thalinger, Empey, Cowperthwaite, & Coveny, 2023) | Mitochondrial 12S region of Birds (260bp) | *Aleadryas rufinucha* (Rufous-naped bellbird) | 2 μl undiluted DNA from swabs and washes, and 2 μl 1:2 diluted DNA from stubs | 1 | 95°C | 10 min |
|  |  |  |  | 39 | 95 °C | 30 sec |
|  |  |  |  |  | 60 °C | 30 sec |
|  |  |  |  |  | 72 °C | 1 min |
|  |  |  |  | 1 | 72 °C | 10 min |
| 16Smam (Taylor, 1996) | Mitochondrial 16S region of Mammals (90bp) | *Balaena mysticetus* (Bowhead whale) or Tasmanian devil (*Sarcophilus harrisii*) | 2μl undiluted DNA | 1 | 95°C | 10 min |
|  |  |  |  | 39 | 95 °C | 12 sec |
|  |  |  |  |  | 59 °C | 30 sec |
|  |  |  |  |  | 70 °C | 25 sec |
|  |  |  |  | 1 | 72 °C | 10 min |
| fwh2 and fwhR2n (Vamos, Elbrecht, & Leese, 2017) | Cytochrome c oxidase 1 (CO1) region of arthropods (205bp) | *Macrotermes belicosus* (Fungus farming termite) and *Eubranchipus grubii* (Brine shrimp) | 2 μl undiluted DNA from swabs and washes, and 2 μl 1:2 diluted DNA from stubs | 1 | 95°C | 5 min |
|  |  |  |  | 35-38 | 95 °C | 30 sec |
|  |  |  |  |  | 58 °C | 30 sec |
|  |  |  |  |  | 72 °C | 45 sec |
|  |  |  |  | 1 | 72 °C | 10 min |
| Trac 01 and ITS-7A (Taberlet, Bonin, Zinger, & Coissac, 2018) | ITS 1 region of vascular plants (267bp) | *Haworthia* spp. | 2μl undiluted DNA | 1 | 95°C | 10 min |
|  |  |  |  | 28 | 95 °C | 30 sec |
|  |  |  |  |  | 56 °C | 30 sec |
|  |  |  |  |  | 72 °C | 30 sec |
|  |  |  |  |  | 72 °C | 10 min |
| 341F and 806R (Sundberg et al., 2013; Yu, Lee, Kim, & Hwang, 2005) | V3-V4 region of bacterial 16S gene (464bp) | N/A | 2μl undiluted DNA | 1 | 95°C | 1 min |
|  |  |  |  | 30 | 95 °C | 15 sec |
|  |  |  |  |  | 56 °C | 15 sec |
|  |  |  |  |  | 72 °C | 30 sec |
|  |  |  |  | 1 | 72 °C | 5 min |
| ITS7F and ITS4ngs (Ihrmark et al., 2012; Tedersoo et al., 2015) | ITS region of Fungi (460bp) | N/A | 2μl undiluted DNA | 1 | 95°C | 1 min |
|  |  |  |  | 30 | 95 °C | 15 sec |
|  |  |  |  |  | 56 °C | 15 sec |
|  |  |  |  |  | 72 °C | 30 sec |
|  |  |  |  | 1 | 72 °C | 5 min |

Table S3: OTU table (Tab 1), full taxa table (Tab 2) and genus-level taxa table (Tab 3) of BirT primer set for Lille Vildmose, Denmark. Table is in a separate excel sheet.

Table S4: OTU table (Tab 1), full taxa table (Tab 2) and genus-level taxa table (Tab 3) of 16Smam primer set for Lille Vildmose, Denmark. Table is in a separate excel sheet.

Table S5: Proportion of samples with genus-level taxonomic detections at the three sampling sites in Denmark (Tab 1) and Ivory Coast (Tab 2). Table is in a separate excel sheet.

Table S6: OTU table (Tab 1), full taxa table (Tab 2) and genus-level taxa table (Tab 3) of fwh (arthropods) primer set for Lille Vildmose, Denmark. Table is in a separate excel sheet.

Table S7: OTU table (Tab 1), full taxa table (Tab 2) and genus-level taxa table (Tab 3) of Trac01 (vascular plant) primer set for Lille Vildmose, Denmark. Table is in a separate excel sheet.

Table S8: Cleaned bacterial ASV table of moss samples from Lille Vildmose, Denmark. Table is in a separate excel sheet.

Table S9: Ecological characterisation of bacterial genera found in moss samples of Lille Vildmose, Denmark. These categorisations were based on online search of each bacterial genus. Table is in a separate excel sheet.

Table S10: Cleaned fungal ASV table of moss samples from Lille Vildmose, Denmark. Table is in a separate excel sheet.

Table S11: OTU table (Tab 1), full taxa table (Tab 2) and genus-level taxa table (Tab 3) of BirT primer set for Lamto Ecological Research Station, Ivory Coast. Table is in a separate excel sheet.

Table S12: OTU table (Tab 1), full taxa table (Tab 2) and genus-level taxa table (Tab 3) of 16Smam primer set for Lamto Ecological Research Station, Ivory Coast. Table is in a separate excel sheet.

**References**

Ihrmark, K., Bodeker, I. T., Cruz-Martinez, K., Friberg, H., Kubartova, A., Schenck, J., . . . Lindahl, B. D. (2012). New primers to amplify the fungal ITS2 region--evaluation by 454-sequencing of artificial and natural communities. *FEMS Microbiol Ecol, 82*(3), 666-677. doi:10.1111/j.1574-6941.2012.01437.x

Sundberg, C., Al-Soud, W. A., Larsson, M., Alm, E., Yekta, S. S., Svensson, B. H., . . . Karlsson, A. (2013). 454 pyrosequencing analyses of bacterial and archaeal richness in 21 full-scale biogas digesters. *FEMS Microbiol Ecol, 85*(3), 612-626. doi:10.1111/1574-6941.12148

Taberlet, P., Bonin, A., Zinger, L., & Coissac, E. (2018). *Environmental DNA: For Biodiversity Research and Monitoring*: Oxford University Press.

Taylor, P. G. (1996). Reproducibility of ancient DNA sequences from extinct Pleistocene fauna. *Mol Biol Evol, 13*(1), 283-285. doi:10.1093/oxfordjournals.molbev.a025566

Tedersoo, L., Anslan, S., Bahram, M., Põlme, S., Riit, T., Liiv, I., . . . Abarenkov, K. (2015). Shotgun metagenomes and multiple primer pair-barcode combinations of amplicons reveal biases in metabarcoding analyses of fungi. *MyoKeys, 10*, 1-43.

Thalinger, B., Empey, R., Cowperthwaite, M., & Coveny, K. (2023). BirT: a novel primer pair for avian environmental DNA metabarcoding. *bioRxiv*. doi:https://doi.org/10.1101/2023.08.08.552521

Vamos, E. E., Elbrecht, V., & Leese, F. (2017). Short COI markers for freshwater macroinvertebrate metabarcoding. *Metabarcoding and Metagenomics, 1*, e14625. doi:https://mbmg.pensoft.net/article/14625/

Yu, Y., Lee, C., Kim, J., & Hwang, S. (2005). Group-specific primer and probe sets to detect methanogenic communities using quantitative real-time polymerase chain reaction. *Biotechnol Bioeng, 89*(6), 670-679. doi:10.1002/bit.20347
